# LSH interacts with and stabilizes GINS4 transcript that promotes tumourigenesis in non-small cell lung cancer

**DOI:** 10.1186/s13046-019-1276-y

**Published:** 2019-06-28

**Authors:** Rui Yang, Na Liu, Ling Chen, Yiqun Jiang, Ying Shi, Chao Mao, Yating Liu, Min Wang, Weiwei Lai, Haosheng Tang, Menghui Gao, Desheng Xiao, Xiang Wang, Fenglei Yu, Ya Cao, Qin Yan, Shuang Liu, Yongguang Tao

**Affiliations:** 1 Department of Pathology, Key Laboratory of Carcinogenesis and Cancer Invasion, Ministry of Education, Xiangya Hospital, Central South University, Changsha, 410078 Hunan China; 2 NHC Key Laboratory of Carcinogenesis of Ministry of Health (Central South University), Cancer Research Institute; School of Basic Medicine, Central South University, Changsha, 410078 Hunan China; 3 Department of Pathology, Xiangya Hospital, Central South University, Changsha, 410008 Hunan China; 4 Department of Thoracic Surgery, Second Xiangya Hospital, Central South University, Changsha, China; 5 Department of Pathology, Yale School of Medicine, New Haven, CT 06520 USA; 6 Department of Oncology, Institute of Medical Sciences, Xiangya Hospital, Central South University, Changsha, 410008 Hunan China

**Keywords:** GINS4, LSH, mRNA stability, Lung cancer

## Abstract

**Background:**

Elucidating mechanisms in oncogenes and epigenetic modifiers are needed to gain insights into the etiology and treatment of cancer, regulation of oncogene by chromatin modifiers at post-transcriptional level is critical and remains unclear. We have investigated the role of GINS4 in NSCLC.

**Methods:**

The expression of chromatin modifier lymphoid-specific helicase (LSH) and GINS4 was assessed in tumor and normal tissue from 79 patients with NSCLC with clinical characteristics. HBE, A549, H358, and H522, PC9, 95C and 95D were cultured after overexpression or silencing of GIAT4RA. Cell proliferation assay, cell migration and invasion assays, plate colony formation assay, immunofluorescence assay, Operetta® high-content screening and analysis, Western blot analysis and Co-Immunoprecipitation (Co-IP) assay, RNA immunoprecipitation assay and tumor growth assay was used to address the potential interplay of between GINS4 and LSH, and the functional of GINS4.

**Results:**

GINS4 is highly expressed in lung cancer cells and tissues, and GINS4 expression is not association with clinical risk factors, but linked with clinical stage and lymphatic metastasis status. Higher expression of GINS4 poorly linked with overall survival in lung adenocarcinomas. Furthermore, GINS4 promoted many characteristics of tumorigenesis including cell growth, clonal formation, migration and invasion, epithelial–mesenchymal transition, tumor sphere and tumor growth in vivo. Interestingly, our results demonstrated that LSH increases GINS4 expression through binding to 3’UTR region of GINS4 and stabilizing its mRNA levels. Finally, LSH overexpression rescues GINS4 knockdown-induced features.

**Conclusions:**

GINS4 facilitates lung cancer progression by promoting key characteristics of tumor potential, and LSH epigenetically interacts with and stabilizes GINS4 transcripts.

**Electronic supplementary material:**

The online version of this article (10.1186/s13046-019-1276-y) contains supplementary material, which is available to authorized users.

## Background

Lung cancer is classified into small cell lung cancer and non-small cell lung cancer (NSCLC), including adenocarcinomas (ADC) and squamous cell carcinoma (SCC), which account for 80–85% of all lung cancer cases [1]. Lung cancer is a leading cause of cancer-related deaths and the most common cancer worldwide, including China, accounting for > 2.8 million deaths in 2015 [2]. Epigenetic modifiers, including chromatin remodeling factors, play important roles in cancer development and progression [1, 3]. Although studies performed on oncogenes and cancer epigenetic factors over the last decade have identified numerous epigenetic modifiers that are involved in the progression of various cancers [4–8], mechanisms underlying the interplay between epigenetic factors and oncogenes in lung cancer remain unclear.

Chromatin modifiers display widespread interactions with RNA transcripts, and regulation of the length of the 3′UTR (3′-untranslated region) is suggested to be associated with the ability of chromatin modifiers to interact with both chromatin and mRNA transcripts [9–12]. SWI/SNF is a large multi-subunit chromatin remodeling complex that can be combinatorially assembled to yield hundreds to thousands of biochemically unique complex and are frequently disrupted in human cancer [13, 14]. Lymphoid-specific helicase (LSH), which belongs to SNF2 family of chromatin-remodeling ATPases, is critical for the normal development of plants and mammals because it establishes correct DNA methylation levels and patterns [15–19]. LSH maintains genome stability in mammalian somatic cells [20, 21]; Moreover, LSH contributes to the malignant progression [22–25]. Our recent study indicated that lncRNA HOTAIR interacts and forms an intact complex with LSH to affect the expression of target genes [26].

Go, Ichi, Nii, and San (means five, one, two, and three, respectively, in Japanese) complex subunit 4 (GINS4) is a member of GINS family of proteins that are essential for the initiation of DNA replication in yeast and Xenopus egg extracts [27, 28]. GINS4 is involved in early embryogenesis in mice and maintains cell cycle progression and genome integrity in *Drosophila* [28, 29], suggesting its role in tumorigenesis. However, the relevance of GINS4 in lung cancer has not been determined to date.

In this study, we examined the physiological role of GINS4 in lung cancer progression and their potential epigenetic mechanisms. We found that LSH increased GINS4 expression by stabilizing its mRNA level post-transcriptionally.

## Material and methods

### Cell culture, antibodies, plasmids, shRNAs and chemicals

Normal lung cell lines, HBE (ATCC: CRL-2741™) were purchased from the ATCC. The lung cancer cell lines A549 (ATCC: CCL-185™), H358 (ATCC: CRL-5807™), and H522 (ATCC: CRL-5810™) were obtained from the ATCC. The lung cancer cell lines PC9, 95C and 95D were obtained from the Cancer Research Institute of Central South University. A549 cells were maintained in DME/F12 1:1(Hyclone), 293 T cells were maintained in DMEM (Gibco), and the other cells were maintained in RPMI 1640 (Gibco). All media were supplemented with 10% (v/v) FBS, and all the cells were maintained at 37 °C in an atmosphere of 5% CO_2_. All the cell lines yielded negative result for mycoplasma contamination. All the cell lines were passaged < 10 times after their initial revival from frozen stocks and were authenticated by performing short tandem repeat profiling before their use.

Actinomycin D, MG132, and CHX were purchased from Selleck (Houston, TX). Vectors overexpressing truncated FLAG–LSH fragments were generated by cloning cDNAs encoding these fragments into pLVX-EF1α-IRES-Puro vector (catalog no. 631988; Clontech, Mountain View, CA) by using restriction enzymes EcoRI and BamHI (Takara). Lentiviral vectors expressing *GINS4* were purchased from Vigene Biosciences (http://www.vigenebio.com; Shandong, China).

Lentiviral shRNA vectors targeting human *GINS4*, and *LSH* and a non-targeting control vector were purchased from Genechem (http://www.genechem.com.cn; Shanghai, China). All the plasmid vectors were verified by performing sequencing.

### Western blot analysis

Western blotting analysis was performed as described previously [30]. Primary antibodies against LSH and α-tubulin were purchased from Santa Cruz Biotechnology, and primary antibody against GINS4 was purchased from GeneTex. EMT Antibody Sampler Kit and primary antibodies against histone H3 were purchased from Cell Signaling Technology, and primary antibody against β-actin was purchased from Sigma-Aldrich (St. Louis, MO).

### Immunohistochemistry (IHC) analysis

Lung cancer tissue samples, which were validated by pathologist Dr. Desheng Xiao (Xiangya Hospital), were obtained from the Department of Pathology of Xiangya Hospital. A lung cancer tissue array was purchased from Pantomics (Richmond, CA). IHC analysis of paraffin-embedded tissue samples obtained from patients with lung cancer was performed as described previously [31].

### Quantitative reverse transcription-PCR and RNA immunoprecipitation assay

qRT-PCR was performed as described previously [30, 31]. Primer sequences used for performing qRT-PCR are as follows: GINS4 forward, 5′-TCAAGCCTGTAATCCCAGCA-3′; GINS4 reverse, 5′-GTTCAAGCGATTCTCCTGCC-3′; β-actin forward, 5′-CACCATTGGCAATGAGCGGTTC-3′; and β-actin reverse, 5′-AGGTCTTTGCGGATGTCCACGT-3′. Results are expressed as mean ± SD of three independent experiments.

RNA immunoprecipitation assay was performed as described previously [32], a total of 10^7^ cells were harvested by trypsinization and resuspended in 2 mL of PBS. The cell lysate was pelleted by centrifugation at 4 °C and 500×*g* for 15 min. The cell lysate was resuspended in 1 mL of RIP buffer, split into three fractions (for Input, Mock, and IP), and then centrifuged at 4 °C and 13,000 rpm for 10 min. Antibodies against normal mouse IgG (Merck Millipore, catalog no. 12–371), normal rabbit IgG (Cell Signaling Technology, catalog no. 2729), and anti-FLAG M2 Magnetic Beads (Sigma Aldrich, catalog no. M8823) were added to the supernatant and incubated overnight at 4 °C with gentle rotation. Next, 40 μL of protein A/G beads were added and the mixture was incubated at 4 °C for an additional hour. The beads were pelleted at 2500 rpm for 30 s, washed three times with 500 μL of RIP buffer and one time with PBS, and then resuspended in 1 mL of RNAiso Plus. The total RNA (input control) and RNA precipitated with the isotype control (IgG) for each antibody were assayed simultaneously with all test samples. The coprecipitated RNAs were detected by qRT-PCR for GINS4.

### Cell proliferation assay, cell migration and invasion assays, plate colony formation assay, immunofluorescence assay, and operetta® high-content screening and analysis

These assays were performed as described previously [30, 31]. Detailed protocols of these assays are mentioned in Additional file 1 Supplementary Material and Methods.

### Oncosphere formation assay

These assays were performed as described previously [33, 34]. Cells were seeded on ultra-low attachment culture dishes (Corning, Corning, NY) in serum-free DMEM-F12 medium containing 50 μg/ml insulin (Sigma-Aldrich St. Louis, MO), 0.4% Albumin Bovine Fraction V (Sigma-Aldrich St. Louis, MO), N^− 2^ Plus Media Supplement (Life Technologies, Grand Island, NY), B-27 Supplement (Life Technologies, Grand Island, NY), 20 μg/ml EGF (PeproTech Rocky Hill, NJ), and 10 μg/ml basic FGF (PeproTech, Rocky Hill, NJ) to support the growth of undifferentiated oncospheres. Cells were incubated in a CO2 incubator for 1–2 weeks, and the numbers of oncosphere cells were counted under a microscope.

### Luciferase assay

To test the regulation of LSH on the GINS4 3′UTR, we constructed a pMiR-REPORT luciferase vector expressing the 3′UTR of GINS4. Luciferase levels were normalized to those of a non-responsive vector expressing Renilla luciferase.

### Nude mice and study approval

The xenograft tumor formation assay was essentially performed as previously described [25, 32–35]. All procedures for animal study were approved by the Institutional Animal Care and Use Committee of the Xiangya School of Medicine of Central South University and confirmed to the legal mandates and federal guidelines for the care and maintenance of laboratory animals. Four-week-old male BALB/c athymic mice were purchased from the Hunan SJA Laboratory Animal Co. Ltd. (http://www.hnsja.com) and housed in dedicated pathogen-free barrier facilities. The mice were injected with the indicated cells in the mammary fat pad. Injected mice were imaged from both the dorsal and ventral sides every 3 days. Data were analyzed using Student’s t-test; a *p* value< 0.05 was considered significant.

### Reproducibility and statistics

All experiments performed in this study were repeated at least three times. Results of western blotting analysis are representative of three independent experiments. All experiments, except experiments involving nude mice, were repeated at least three times.

Results are expressed as mean ± SD or SEM, as indicated. A two-tailed Student’s t-test was used for performing intergroup comparisons. A *p* value of < 0.05 was considered statistically significant. *, **, and *** indicate *P* < 0.05, *P* < 0.01, and *P* < 0.001, respectively.

## Results

### GINS4 is highly expressed in lung cancer tissues and is associated with the poor survival of patients with lung ADC

**Fig. 1:**
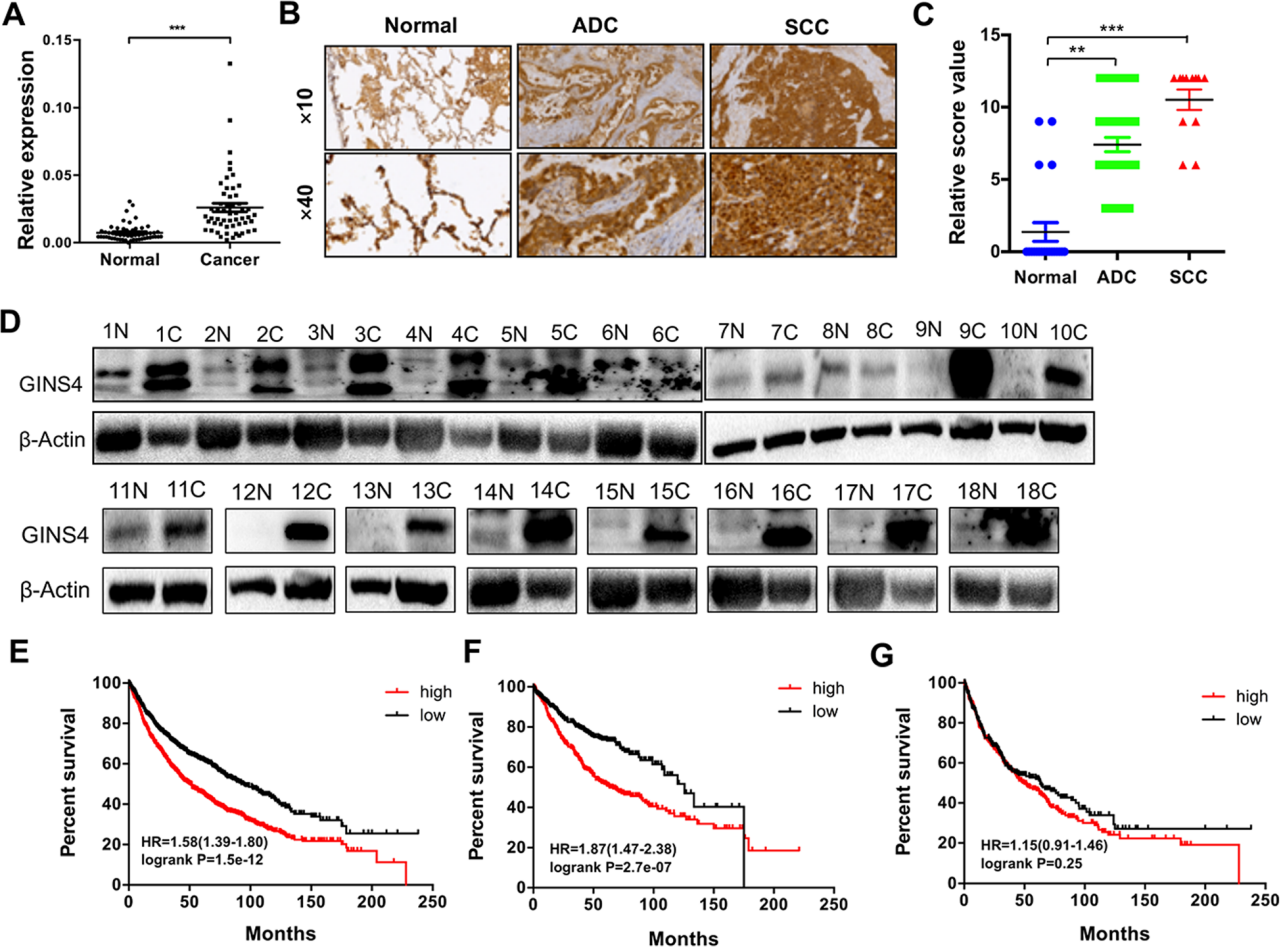
GINS4 is highly expressed in lung cancer tissues and is associated with the poor survival of patients with lung ADC. **a** qRT-PCR analysis of GINS4 expression in 79 lung cancer tissue samples and corresponding paired normal lung tissue samples. **b** and **c** IHC analysis was performed to determine GINS4 protein level in lung cancer and normal lung tissue samples (**b**). Mean values of IHC quantification of GINS4 protein level are shown for lung cancer tissue samples compared with those for normal lung tissue samples (**c**). **d** Western blotting analysis of GINS4 in 11 lung ADC cancer and normal lung tissue samples. **e-g** Kaplan–Meier curves for the overall survival rates of patients with (**e**) lung cancer, (**f**) lung ADCs, and (**g**) lung SCCs. **P < 0.01 and ***P < 0.001

We previously found that LSH upregulated *GINS4* mRNA expression using RNA sequencing [36]. Here we first determined the role of GINS4 in patients with lung cancer, we performed qRT-PCR analysis of an independent panel of 79 primary NSCLC tissues and normal lung tissues. GINS4 expression was upregulated in the primary lung cancer tissues (Fig. 1a). To further determine GINS4 expression level in lung cancer, we performed IHC analysis of tissues obtained from patients with lung cancer. GINS4 protein was detected in the cytoplasm and nucleus of cells isolated from normal lung tissues, and its expression was highly increased in both lung ADC and SCC tissues (Fig. 1b-c). Furthermore, TCGA database indicated that GINS4 was highly expressed in both lung ADC and SCC tissues (Additional file 1: Figure S1A-B). Moreover, GINS4 protein levels were clearly increased in lung cancer tissues obtained from cases 1–10 in Fig. 1d with lung ADCs, and cases 11–18 of Fig. 1d with lung SCCs.

Finally, multivariate analysis showed that the expression level of GINS4 was independent of clinical risk factors such as gender, smoking, tumor differentiation and tumor size, but linked with clinical stages and lymphatic metastasis (Table 1). Kaplan–Meier analysis of these patients showed that GINS4 expression was associated with poor overall survival in all lung cancers (Fig. 1e), and lung ADCs (Fig. 1f), but not in lung SCCs (Fig. 1g). Together, these findings indicate that GINS4 is highly expressed in lung cancer and suggest that it functions as an oncogene in lung cancer progression.

**Table 1:** GINS4 expression level and Main characteristics of the patients (*N* = 79)

Characteristics	*n* (%)	Relative expression level (Mean)	*P* value
Gender			
Male	55 (70)	0.024	0.273
Female	24 (30)	0.027	
Smoking History			
Yes	41 (52)	0.028	0.280
No	38 (48)	0.024	
Histology			
ADC	49 (62)	0.028	0.359
SCC	30 (38)	0.023	
Differentiation			
Poor and moderate	70 (89)	0.029	0.115
Well	9 (11)		
T stage			
T1	18 (23)	0.021	0.004
T2	59 (75)	0.029	
T3	5 (6)	0.041	
N stage			
N0	44 (56)	0.020	0.002
N1-N3	35 (44)	0.033	
Clinical Stages			
I-II	58 (73)	0.023	< 0.001
III-IV	21 (27)	0.036	

### LSH induces GINS4 expression and binds with GINS4 transcript

**Fig. 2:**
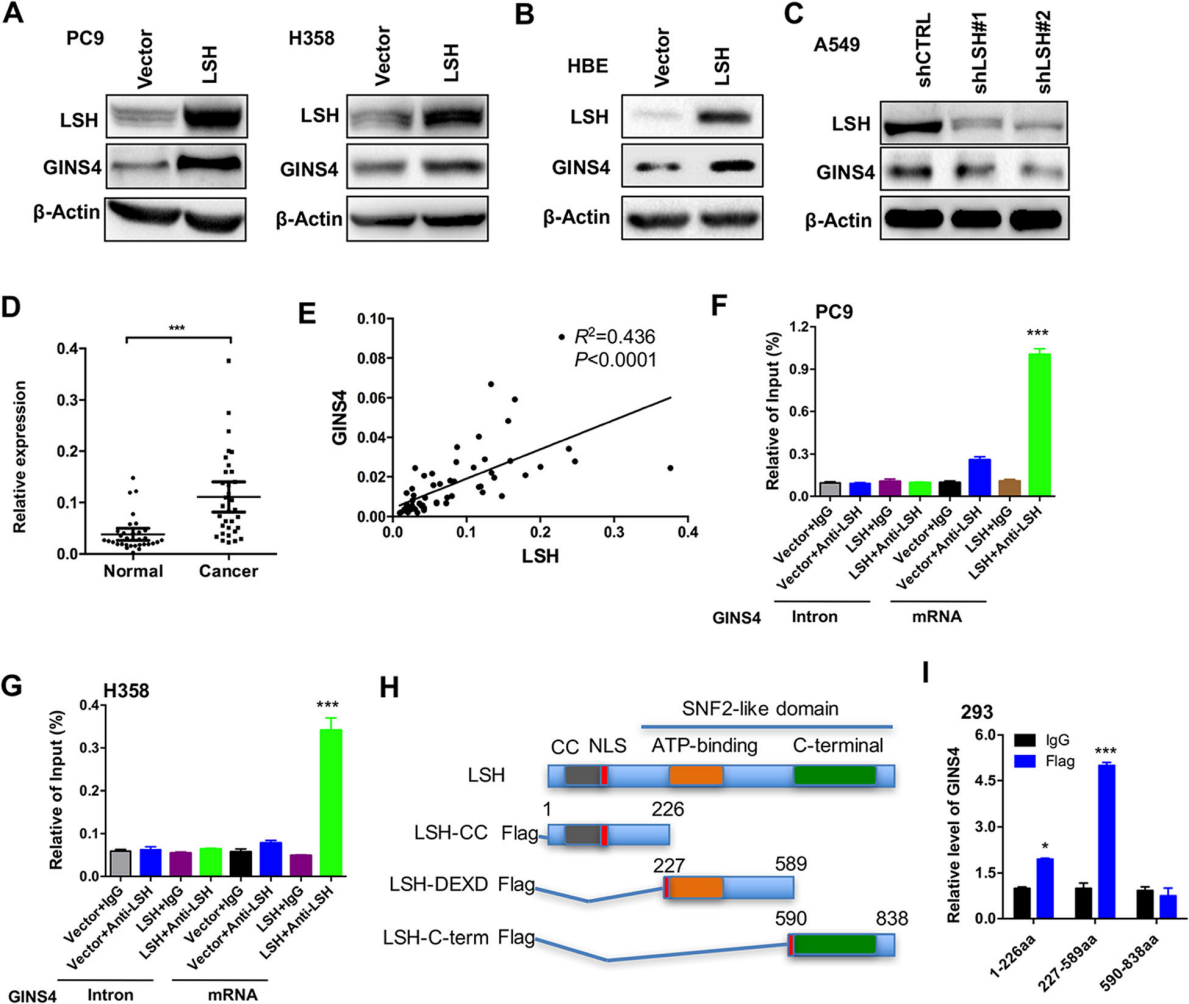
LSH induces GINS4 expression and binds with GINS4 transcript. **a-b** Western blotting analysis of LSH and GINS4 in LSH-overexpressing (**a**) PC9 and H358, and HBE (**b**) cells (*n* = 3). **c** Western blotting analysis of LSH and GINS4 in LSH-depleted A549 cells (*n* = 3). **d** qRT-PCR analysis of LSH expression in 79 lung cancer tissue samples and corresponding paired normal lung tissue samples. **e** Correlation between LSH and GINS4 expression was analyzed. (F-G) qRT-PCR analysis was performed to determine the relative binding of LSH to the GINS4 mRNA in LSH-overexpressing (**f**) PC9 and (**g**) H358 cells and in (**h-i**) 293 cells overexpressing truncated LSH fragments (**h**). **i** Results were normalized using those obtained for respective IgG controls, with the 18S rRNA gene as a loading control (*n* = 3)

Next, we performed western blotting analysis to show that LSH overexpression promoted GINS4 protein expression in H358, PC9 and HBE cells (Fig. 2a-b). In addition, stable LSH knockdown decreased GINS4 protein expression in A549 cells (Fig. 2c), suggesting the regulatory role of LSH in GINS4 expression *in cis*. Consistent with our previous findings, we found that LSH was highly expressed in lung cancer tissues (Fig. 2d) and was positively correlated with GINS4 expression (Fig. 2e).

Chromatin modifiers might interact with both chromatin and mRNA transcripts [9–11], indicating that chromatin modifiers are involved in the post-transcriptional regulation. To determine whether LSH protein binds to GINS4 mRNA, we performed RNA immunoprecipitation (RIP) assay by using an anti-LSH antibody. We found that LSH was recruited to the *GINS4* mRNA in PC9-LSH and H358-LSH cells but was not recruited to the intron region of the *GINS4* mRNA (Fig. 2f-g).

Next, we constructed a series of vectors expressing truncated LSH fragments (LSH fragment lacking the N-terminal domain and containing a coiled-coil domain [1–226 AA], LSH fragment containing an ATP-binding domain [227–589 AA], and LSH fragment containing C-terminal region of SNF2 domain [590–838 AA]; Fig. 2h). Analysis by using a series of truncated LSH fragments showed that both 1–226 AA and 227–589 AA of LSH interacted with *GINS4* mRNA (Fig. 2i), indicating that LSH binding directly induced changes in *GINS4* mRNA expression and that the ATP-binding domain of LSH was mainly critical for the binding of LSH to *GINS4* mRNA.

### LSH increases GINS4 stabilization through 3’UTR of GINS4

**Fig. 3:**
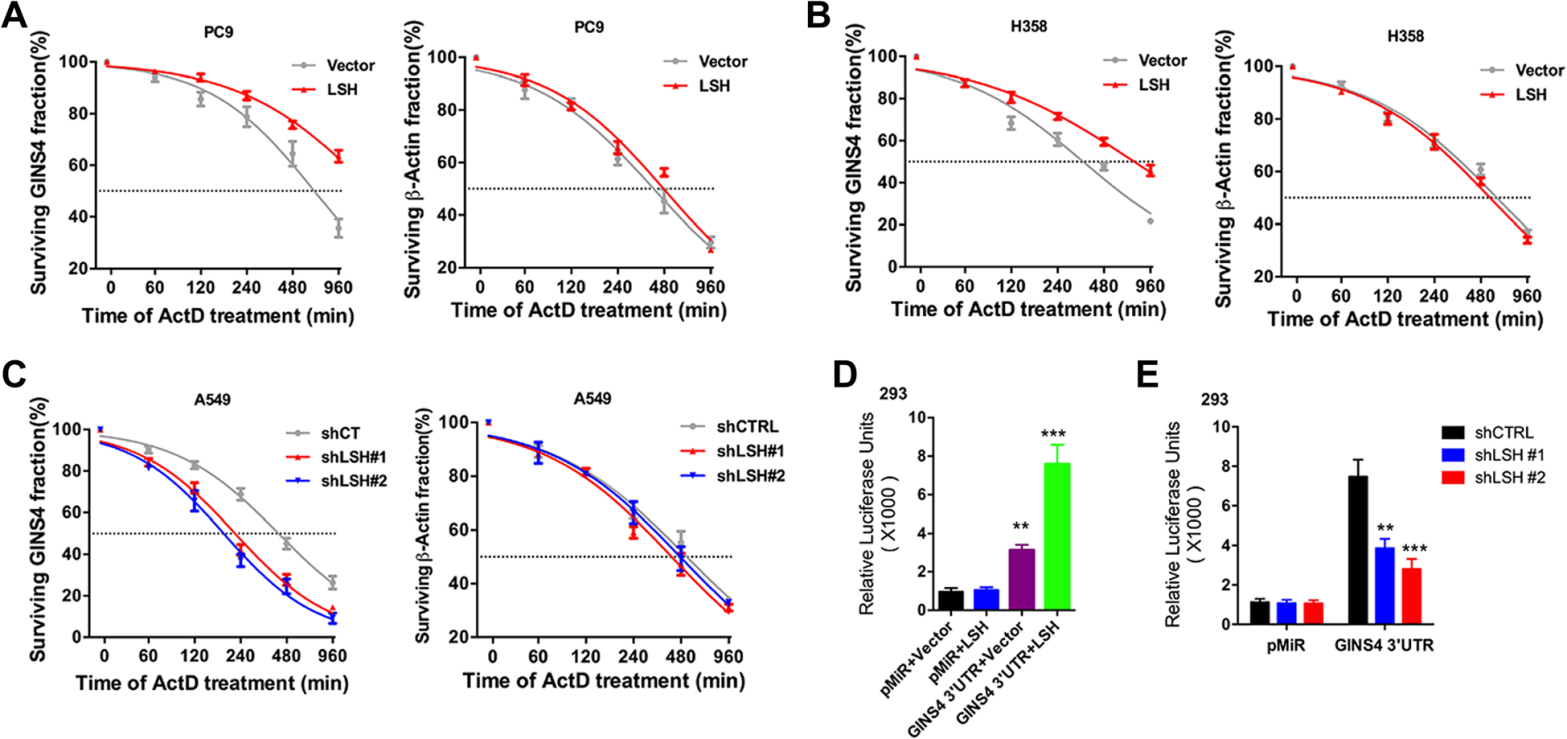
LSH increases GINS4 stabilization through 3’UTR of GINS4. **a-c** LSH-overexpressing (**a**) PC9 and (**b**) H358 cells and (**c**) *LSH*-knockout A549 cells were treated with actinomycin D (5 μg/mL) for the indicated time. *GINS4* and β-actin mRNA stability was assayed by performing qRT-PCR, with the 18S rRNA gene as a loading control (*n* = 3). **d** Luciferase activity in 293 cells coexpressing a luciferase reporter vector encoding the *GINS4* 3′UTR and LSH (*n* = 4). **e** Luciferase activity in LSH-depleted A549 cells expressing a luciferase reporter vector encoding the *GINS4* 3′UTR (*n* = 4). *P < 0.05, **P < 0.01, and ***P < 0.001

The treatment of LSH-overexpressing cells with a transcriptional inhibitor actinomycin D [37] showed that LSH overexpression significantly increased the half-life of *GINS4* mRNA by 2.4 fold but did not affect the stability of β-actin mRNA in PC9 cells (Fig. 3a). LSH overexpression also significantly increased the half-life of *GINS4* mRNA by two fold but did not affect the stability of β-actin mRNA in H358 cells (Fig. 3b). However, LSH depletion significantly decreased the half-life of *GINS4* mRNA by two fold in A549 cells (Fig. 3c). Results of reporter assays showed an increase in luciferase activity in 293 cells coexpressing full-length *GINS4* 3′UTR (Luc + 3′UTR) and LSH (Fig. 3d). This may be because of an increase in GINS4’s regulation of LSH through its 3′UTR. This regulatory induction was decreased after LSH depletion in A549 cells (Fig. 3e). Together, these findings indicate that LSH increases GINS4 expression and stabilization through directly binding.

### Overexpression of GINS4 promotes cancer cell growth, migration and invasion

To address the role of GINS4 in lung cancer, we first performed western blotting analysis to determine GINS4 expression in a panel of lung cells and found that GINS4 expression was higher in lung cancer cells than in normal lung cells (Additional file 1: Figure S2A). Results of qRT-PCR confirmed that GINS4 expression was higher in lung cancer cells than in normal lung cells (Additional file 1: Figure S2B). Western blotting analysis of the nuclear and cytoplasmic fractions of H1299, 95C, PC9, A549, SPCA-1 and HBE cells showed that majority of GINS4 was present in the nucleus of these cells (Additional file 1: Figure S2C). Next, we selected HBE, A549, PC9, and H1299 cells to determine the role of GINS4 in lung cancer progression.

**Fig. 4:**
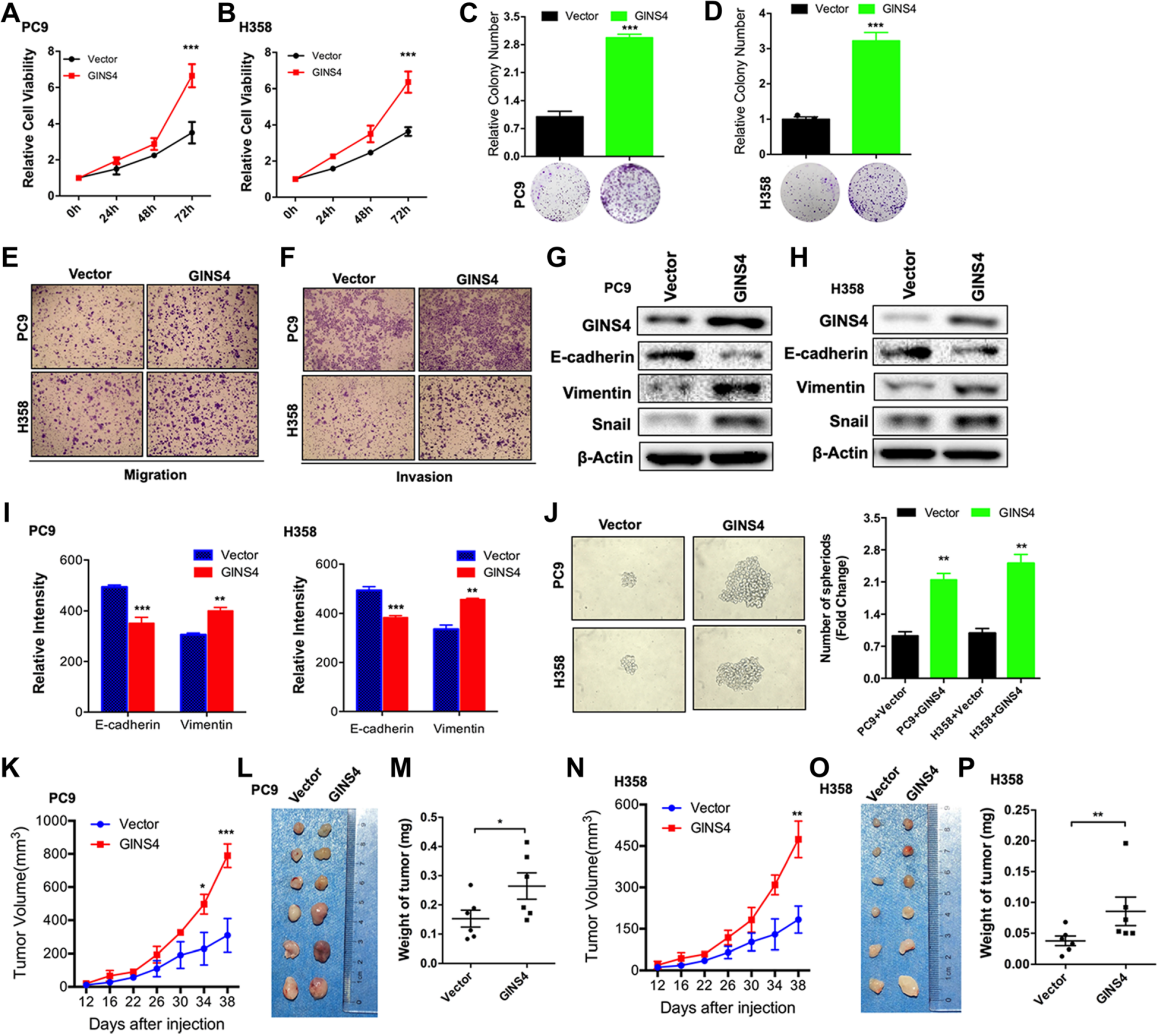
Overexpression of GINS4 promotes cancer cell growth, migration and invasion. **a** and **b** MTT assay was performed to assess the viability of (**a**) PC9 and (**b**) H358 lung cancer cells overexpressing GINS4 (*n* = 3). **c** and **d** Plate colony formation assay was performed to determine the colony formation ability of GINS4-overexpressing (**c**) PC9 and (**d**) H358 cells. Relative colony number is shown as a bar graph (mean ± SD of three separate experiments) in the upper region, and a representative image of the colony number is shown in the lower region. **e** and **f** A representative image showing the migration (**e**) and invasion (**f**) of GINS4-overexpressing PC9 and H358 cells (*n* = 3). **g** and **h** Western blotting analysis detected EMT-related proteins in GINS4-overexpressing (**g**) PC9 and (**h**) H358 cells (*n* = 3). **i** High-content screening and analysis were performed to detect the intensity of E-cadherin and vimentin staining in PC9 (left) and H358 (right) cells stably expressing a control vector or GINS4 (*n* = 4). **j** The GINS4 expressing sublines and control cells were plated in ultra-low-attachment dishes to allow tumor sphere formation (left). The fold changes of spheres formed by both the GINS4 expressing cells and control cells are shown in the right panel (*n* = 3). **k-m** A xenograft nude mouse model was established, and the volume of tumors derived from GINS4-overexpressing PC9 cells or control cells was determined at indicated time points (**k**). Images showing the size (**l**) and weight (**m**) of tumors isolated from nude mice injected with GINS4-expressing PC9 cells or control cells. **n-p** A xenograft nude mouse model was established, and the volume of tumors derived from GINS4-overexpressing PC9 cells or control cells was determined at indicated time points (**n**). **o** and **p** Images showing the size (**o**) and weight (**p**) of tumors isolated from nude mice injected with GINS4-expressing H358 cells or control cells. *P < 0.05, **P < 0.01, and ***P < 0.001

We first stably overexpressed GINS4 in lung cancer cell lines PC9 and H358, and we also found that GINS4 did not affect LSH expression (PC9-GINS4 and H358-GINS4, respectively; Additional file 1: Figure S2D). GINS4 overexpression significantly increased the growth of these cells in vitro (Fig. 4a-b) and enhanced their colony formation ability (Fig. 4c-d). Moreover, stable GINS4 expression increased the migration and invasion of PC9 and H358 cells in vitro (Fig. 4e-f). Furthermore, stable GINS4 expression impaired the expression of epithelial marker E-cadherin in PC9 cells and in H358 cells and increased the expression of mesenchymal markers Vimentin and Snail (Fig. 4g-h), suggesting that GINS4 promoted epithelial–mesenchymal transition (EMT) in these cells. Analysis by using the high-content imaging system showed that GINS4 overexpression decreased the relative intensity of E-cadherin staining and increased the relative intensity of vimentin staining in both PC9 and H358 cells (Fig. 4i). Next, we found that the GINS4 expressing cells formed larger and more abundant tumor spheres that the control cells in both PC9 and H358 cells through tumor sphere assays (both ** *P* < 0.01) (Fig. 4j).

To determine whether GINS4 also played a role in lung cancer progression in vivo, we established a xenograft nude mouse model and examined xenograft tumor formation in this model. We found that nude mice injected with PC9-GINS4 cells formed tumors of significantly larger tumor volume, tumor size and tumor weight than nude mice injected with control cells (Fig. 4k-m). The injection of H358-GINS4 cells (1 × 10^7^) also showed that GIINS4 overexpression significantly increased the tumor volume, tumor size, and tumor weight (Fig. 4n-p). Furthermore, GINS4 accelerated EMT through Vimentin and E-cadherin staining after overexpression of GINS4 in PC9 and H358 biopsies compared with that in the control cells biopsies from xenograft tumors. However, GINS4 did not change LSH expression according to IHC in LSH in PC9 and H358 cells biopsies among the three biopsies (Additional file 1: Figure S3). Together, these results indicate that GINS4 overexpression promotes the growth, migration, and invasion of lung cancer cells.

### *GINS4* knockdown inhibits cancer progression in vitro

**Fig. 5:**
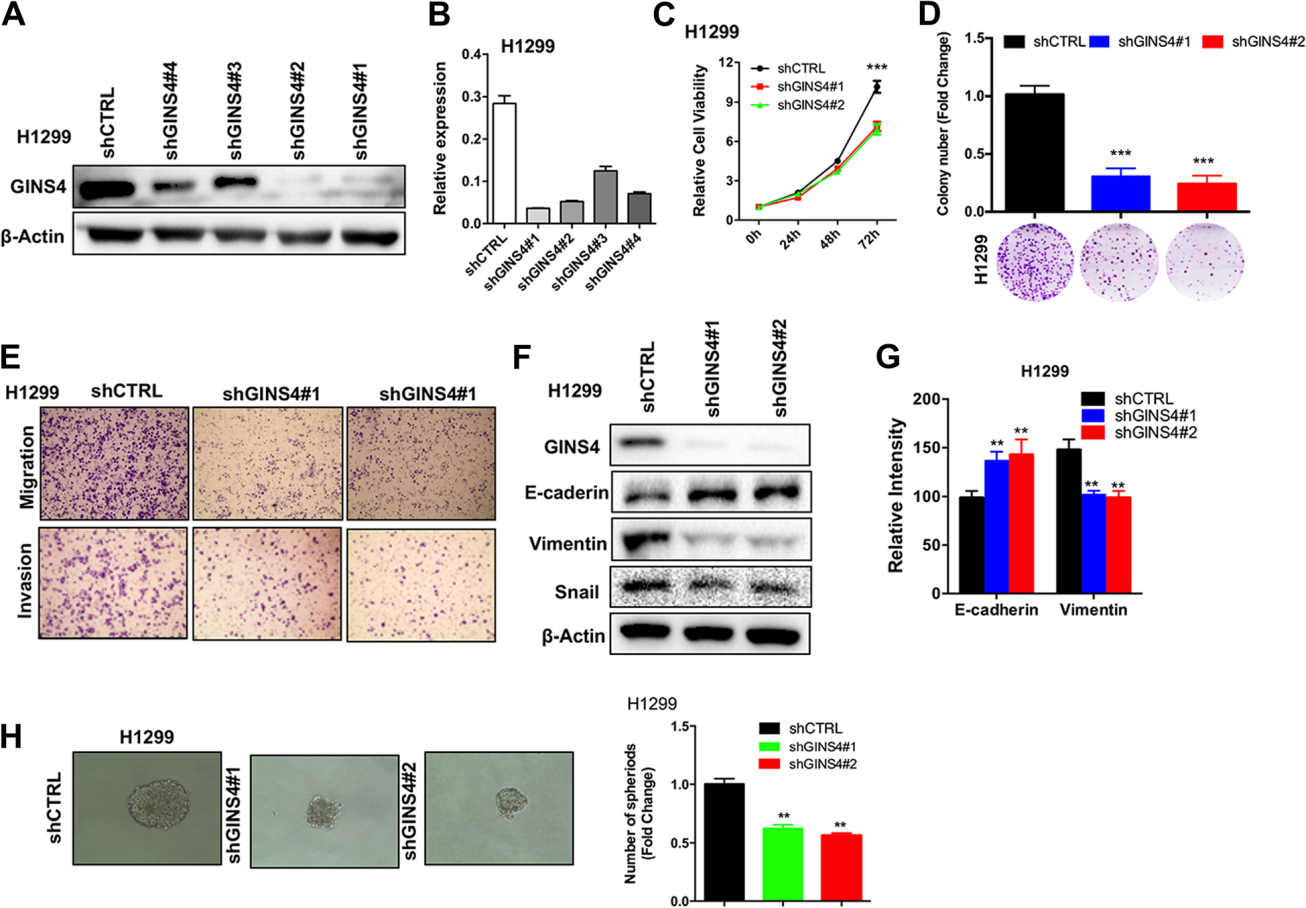
*GINS4* knockdown inhibits lung cancer migration and invasion in vitro. **a** and **b** H1299 cells were stably transfected with four distinct *GINS4* shRNA expression vectors (shGINS4#1–shGINS4#4) or a control vector (shCTRL). GINS4 expression levels determined by performing (**a**) western blotting analysis and (**b**) qRT-PCR are shown. **c** The MTT assay was performed to assess the viability of H1299 cells stably transfected with two distinct *GINS4* shRNA expression vectors (shGINS4#1 and shGINS4#1) or the control vector (shCTRL) (*n* = 3). **d** Plate colony formation assay was performed to measure the colony formation ability of GINS4-depleted H1299 cells. Relative colony number is shown as a bar graph (mean ± SD of three separate experiments) in the upper region, and a representative image of the colony number is shown in the lower region (*n* = 3). **e** A representative image showing the migration (upper region) and invasion (lower region) of stable *GINS4*-knockout H1299 cells (*n* = 3). **f** Western blotting analysis detected EMT-related proteins in GINS4-depleted H1299 cells (*n* = 3). **g** High-content screening and analysis were performed to detect the intensity of E-cadherin and vimentin staining in GINS4-depleted H1299 cells (*n* = 4). **h** The GINS4-depleted H1299 cells or control cells were plated in ultra-low-attachment dishes to allow tumor sphere formation (left). The fold changes of spheres formed by both the GINS4-depleted H1299 cells or control cells are shown in the right panel (*n* = 3). * *P* < 0.05, ** *P* < 0.01 and *** *P* < 0.001

To further validate the role of GINS4 in lung cancer progression, we generated stable *GINS4*- knockdown H1299 cells. All *GINS4*-targeting shRNAs reduced *GINS4* mRNA and protein levels in H1299 cells; moreover, *GINS4* knockdown by using shGINS4#1 and shGINS4#2 successfully reduced *GINS4* mRNA and protein levels to < 10% (Fig. 5a-b). *GINS4* knockdown significantly reduced the growth of H1299 (Fig. 5c). Moreover, *GINS4* knockdown impaired the colony formation ability (Fig. 5d) and reduced the migration and invasion abilities of H1299 cells (Fig. 5e). Moreover, stable *GINS4* knockdown increased the relative intensity of E-cadherin and impaired the relative intensity of Vimentin and Snail staining in H1299 cells (Fig. 5f). Analysis by using the high-content imaging system showed that *GINS4* knockdown increased E-cadherin staining and attenuated vimentin expression in H1299 cells (Fig. 5g). Next, we demonstrated that depletion of GINS4 formed smaller and less abundant tumor spheres that the control cells in H1299 cells through tumor sphere assays (both ** *P* < 0.01) (Fig. 5h). Together, these results indicate that depletion of GINS4 inhibits the growth, migration, and invasion of lung cancer cells.

### *GINS4* knockdown inhibits cancer progression in vivo

**Fig. 6:**
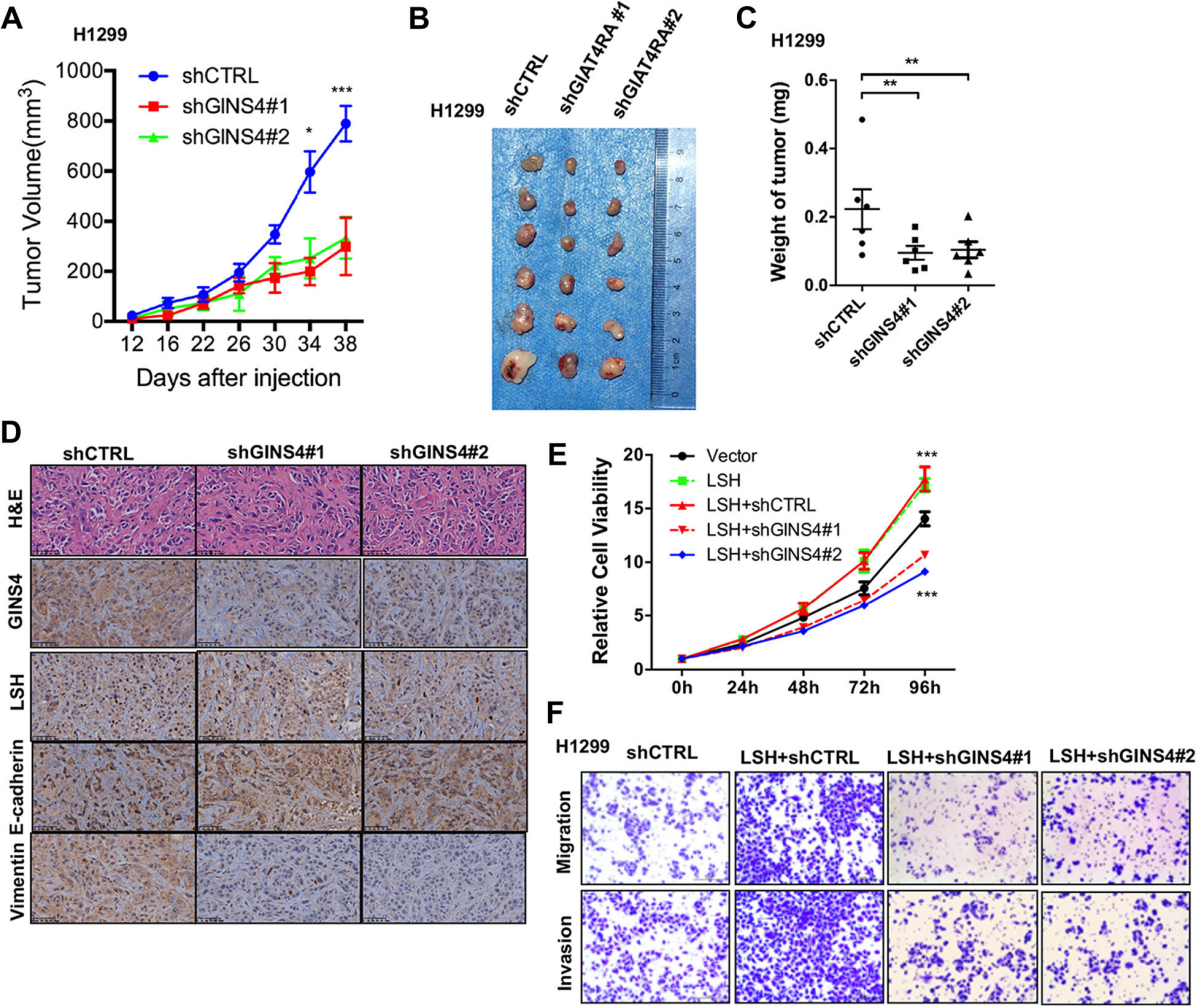
*GINS4* knockdown inhibits lung cancer migration and invasion in vitro. **a-c** A xenograft nude mouse model was established, and volumes of tumors derived from *GINS4*-knockout H1299 cells or control cells were determined at indicated time points (**a**). Images showing the size (**b**) and weight (**c**) of tumors isolated from nude mice injected with GINS4-depleted PC9 cells or control cells. **d** Hematoxylin and Eosin staining and immunohistochemistry staining for the indicated proteins of a representative primary xenograft originating from GINS4-depleted H1299 cells or control cells. Original magnification 400 ×, scale bar, 50 μm. **e** The MTT assay was performed to assess the viability of LSH-overexpressing and GINS4-depleted H1299 cells. **f** A representative image showing the migration (upper region) and invasion (lower region) of LSH-overexpressing and GINS4-depleted H1299 cells (*n* = 3). * *P* < 0.05, ** *P* < 0.01 and *** *P* < 0.001

To further determine whether GINS4 also played a role in lung cancer progression in vivo, we injected 3 × 10^6^ *GINS4*- knockdown H1299 cells into nude mice and found that *GINS4* knockdown significantly decreased the volume, size and weight of tumors derived from these cells (Fig. 6a-c). However, no significant change was observed in the body weights of mice injected with *GINS4*-knockdown H1299 cells and control cells. Furthermore, depletion of GINS4 impaired EMT through Vimentin and E-cadherin staining after depletion of GINS4 in H1299 biopsies compared with that in the control cells biopsies from xenograft tumors. However, GINS4 did not change LSH expression in H1299 cells after depletion of GINS4 biopsies among the three biopsies (Fig. 6d). Together, these findings indicate the physiological role of GINS4 in the growth, migration, and invasion characteristics of lung cancer cells.

To further address whether GINS4 promotes tumorigenic potential under the control of LSH, we detected cell growth of lung cancer cells after LSH was transfected into the GINS4-depleted cells. We found that LSH overexpression-induced growth proliferation of H1299 cells was attenuated after depletion of GINS4 (Fig. 6e). Moreover, we showed that the increase of LSH in migration and invasion abilities of H1299 cells was attenuated after depletion of GINS4. Taken together, GINS4 accelerates tumorigenic potential dependent of LSH expression.

## Discussion

In the present study, we found that GINS4 facilitates lung cancer progression by promoting cancer cell growth, migration, and invasion, which are the key characteristics of cancer progression. Epigenetic regulation involves in the regulation of GINS4, LSH upregulates GINS4 by stabilizing *GINS4* mRNA level.

LSH is critical for DNA methylation because it functions as a chromatin silencer [16, 17, 38, 39]. Moreover, LSH increases nucleosome density [39], induces RNA polymerase II stalling [40, 41] and promotes gene silencing through a G9a–GLP complex during differentiation and early development [18]. Moreover, LSH contributes to carcinogenesis as a transcriptional repressor [22, 23, 25, 36, 42, 43]. Interestingly, we found that LSH overexpression upregulated GINS4 expression independently of DNA methylation. We further firstly provide evidence for the increased role of LSH in gene expression through mRNA stability instead of silencing, indicating that LSH involves the target genes in a selective manner. Our findings further indicate that chromatin modifiers, including histone methyltransferases, post-transcriptionally regulate mRNA levels [9–11, 44].

GINS4 complexes with other moieties such as PSF1, PSF2, and PSF3 to promote DNA replication in yeast [27, 28, 45]. High expression of GINS genes such as *PSF1* has been detected in different cancers, and expression levels of GINS genes are suggested to be correlated with growth retardation, cell cycle arrest, malignancy, and stem cell-like properties; for example, high *PSF1* promoter activity has been detected in cancer-initiating cells or cancer stem cells in a murine tumor cell transplantation model [46, 47]. High expression levels of GINS genes may induce cell growth as well as chemotherapy resistance. Interestingly, another replication licensing factor Cdc6 undergoes phenotypic changes with mesenchymal features and loss of E-cadherin by direct binding to the promoter beyond activating adjacent replication origins [48]. In the present study, we analyzed the functions of GINS4 besides DNA replication and found that GINS4 promoted EMT, migration, invasion, and metastasis in lung cancer. High expression levels of PSF1 and PSF2 but not of PSF3 are associated with the poor survival of patients with lung cancer and ADC but not the survival of patients with SCC (Additional file 1: Figure S4). The reasons for this is possible that the driver genes in SCCs and ADCs are different [49].

Interestingly, high expression of GINS4 is associated with the poor survival of patients with lung ADC and gastric cancer, but not the survival of patients with lung SCC and breast cancer (Additional file 1: Figure S5), indicating that GISN4 functions as an oncogene depending on the cancer type.

## Conclusions

In summary, our study highlights the importance of GINS4 in lung cancer migration, invasion, and progression. LSH increased GINS4 protein level by increasing the stability of the *GINS4* mRNA. GINS4 as well as other GINS family members have the potential to regulate cancer cell proliferation, suggesting their use as therapeutic targets for cancer treatment. Our findings suggest that the LSH–GINS4 axis can be used as a potential target for developing novel therapeutic approaches.

## Additional files

Additional file 1: Expression of GINS4 in various malignant tumors and its relationship with prognosis. (DOC 13705 kb)

## Data Availability

Not applicable.

## Abbreviations

3′UTR.: 3′-untranslated region
ADC: Adenocarcinomas
Co-IP: Co-Immunoprecipitation
EMT: Epithelial–mesenchymal transition
GINS4: Go, Ichi, Nii, and San (means five, one, two, and three, respectively, in Japanese) complex subunit 4
IHC: Immunohistochemistry
LSH: Lymphoid-specific helicase
NSCLC: Non-small cell lung cancer
RIP: RNA immunoprecipitation
SCC: Sequamous cell carcinoma
