# Supplementary material for: LSH interacts with and stabilizes GINS4 transcript that promotes tumourigenesis in non-small cell lung cancer
Source: J Exp Clin Cancer Res. 2019 Jun 28;38:280. doi: 10.1186/s13046-019-1276-y (PMC6599244; doi:10.1186/s13046-019-1276-y)
Supplement: Supplementary file 1 — Expression of GINS4 in various malignant tumors and its relationship with prognosis. (DOC 13705 kb) [file 13046_2019_1276_MOESM1_ESM.doc]

**LSH interacts with and stabilizes GINS4 transcripts that promotes tumourigensis in non-small cell lung cancer**

**Supplementary Material and Methods**

**Lentiviral infection and transient transfection**

To generate knockdown GINS4 cells, GV248-shGINS4 lentivirus plasmid was transfected into 293T cells with psPAX2 and pMD2.G. Viral supernatant factions were collected at 48 hours after transfection and filtered through a 0.45μm filter followed by infection into H1299 cells together with 5μg/ml polybrene and incubation for 16 h. At 48 hours after infection, replace with fresh medium with 1μg/ml puromycin.

To generate knockdown LSH cells, pLJM1-Mock-shLSH lentivirus plasmid was transfected into 293T cells with pCMV-VSV-G, pMDLg-pRRE and pRSV-Rev (Addgene). Viral supernatant factions were collected at 48 hours after transfection and filtered through a 0.45μm filter followed by infection into A549 cells together with 5μg/ml polybrene and incubation for 16 hours. At 48 hours after infection, replace with fresh medium with 1μg/ml puromycin and incubated for another 6 days.

To generate stable over-expression LSH cells, plvx-LSH lentivirus plasmid was transfected into 293T cells with pCMV-VSV-G, pMDLg-pRRE and pRSV-Rev (Addgene). Viral supernatant factions were collected at 48 hours after transfection and filtered through a 0.45μm filter followed by infection into H358 and PC9 cells together with 5μg/ml polybrene and incubation for 16 hours. At 48 hours after infection, replace with fresh medium with 1μg/ml puromycin and incubated for another 6 days.

To generate over-expression GINS4 cells, pEnter-GINS4 plasmid was transfected into H358 and PC9 cells. At 48 hours after transfection, replace with fresh medium with 1μg/ml puromycin and incubated for another 6 days.

**Quantitative real-time PCR**

Cells were harvested with Trizol (Invitrogen). cDNAs were synthesized with SuperScript III (Invitrogen) according to the manufacturer’s protocol. Real-time PCR analysis was performed using the Applied Biosystems 7500 Real-Time PCR System, according to the manufacturer’s instructions. The reactions were performed in triplicates for three independent experiments: the results were normalized to **β**-actin. The mean± SD of three independent experiments was shown.

**Cell proliferation assay, migration and invasion assay and plate-colony formation assay.**

Details of the cell proliferation assay were described previously (1). For plate-colony formation assay, cells (3×10^3^/mL/well) were seeded were plated in 96-well plates. After [incubation](https://www.sciencedirect.com/topics/medicine-and-dentistry/incubation), 20 μL of 3-(4,5-dimethylthiazol-2-yl)-5-(3-carboxymethoxyphenyl)-2-(4-sulfophenyl)- 2H-tetrazolium (MTS, Promega) was added to each well and incubated at 37 °C for 4 h. [Absorption](https://www.sciencedirect.com/topics/biochemistry-genetics-and-molecular-biology/absorption) was determined at 490 nm on a multiple-well plate reader (Bio-Tek).

The migration and invasion assays were described previously (2). Cells (5 × 10^5^) were seeded onto the upper chamber in 200 μL of serum-free medium; the lower compartment was filled with 0.6 mL of DMEM media supplemented with 10% of FBS. After 24 hours incubation, migrated cells on the lower surface of the filter were fixed and stained using propidium iodide. Cells on the upper side were removed using a rubber scraper. Fluorescent images were obtained. The reported data represents counts of migrated cells. Experiments were performed in triplicates.

For the plate-colony formation assay, cells (2 × 10^3^/ml/well) were seeded into 6-well plates and cultured in RPMI-1640 medium supplemented with 10% FBS. Colonies were fixed with methanol, stained with viola crystallina and scored using a microscope and ImageJ software (1.47V, NIH, USA).

**RNA immunoprecipitation assays**

10^7^ cells were harvested by trypsinization and resuspended in 2 ml of PBS. The cell lysate was pelleted by centrifugation at 4°C and 500 g for 15 min. The cell lysate was resuspended in 1 ml of RIP buffer (150 mM KCl, 25 mM Tris at pH 7.4, 5 mM EDTA, 0.5 mM DTT, 5% NP40, 9 μg/ml leupeptin, 9 μg/ml pepstatin, 10 μg/ml chymostatin, 3 μg/ml aprotinin, 1 mM PMSF, and 100 U/ml RNasin Ribonuclease Inhibitors (Promega)). The suspended lysate was split into three fractions, 500 μl each (for Input, Mock and IP), and the lysate was subjected to centrifugation at 4°C and 13,000 rpm for 10 min. The antibodies against normal mouse IgG (Merck Millipore, cat.#12-371), normal rabbit IgG (Cell Signaling Technology, cat# 2729), and Anti-FLAG M2 Magnetic Beads (Sigma Aldrich, cat# M8823) were added to supernatant and incubated overnight at 4°C with gentle rotation. 40 μl of protein A/G beads were added, and the mixture was incubated for 1 hr at 4°C with gentle rotation. The beads were pelleted at 2,500 rpm for 30 sec. The supernatant was removed, and the beads were resuspended in 500 μl of RIP buffer. This was repeated for a total of 3 RIP washes and followed by 1 wash in PBS. Then, the beads were resuspended in 1 ml of RNAiso Plus. The total RNA (input control) and precipitation with the isotype control (IgG) for each antibody were assayed simultaneously. The co-precipitated RNAs were detected by qRT–PCR for GIAT4RA (Forward, 5’-TTGGTGGATGCTGACCTTCA-3’ left 5’-ACCAGAAACCAGATGTAGCCA-3’).

**Nude mice and study approval**

A xenograft tumor formation was essentially performed as previously described (2). SCID Mice (Hunan SJA Laboratory Animal Co.，Ltd.) were injected with A549 (1 × 10^6^ cells/mouse) or H358 (2 × 10^6^ cells/mouse) cells via mammary fat pad (10 mice/group). Mice with A549 or H358 cells were imaged from dorsal and ventral views every three days. Data were analyzed using Student’s t-test; a *p* value< 0.05 was considered significant.

All procedures for animal study were approved by the Institutional Animal Care and Use Committee of the Central South University of Xiangya School of Medicine and confirm to the legal mandates and federal guidelines for the care and maintenance of laboratory animals.

**Immunohistochemistry (IHC) analysis**

Lung and related diseases biopsies were validated and obtained from Department of Pathology in Xiangya Hospital. IHC analysis of paraffin sections from lung cancer tissues was described previously (2). The sections were incubated with antibodies as indicated. The images were surveyed and captured using a CX41 microscope (OLYMPUS, Tokyo, Japan) with the Microscope Digital Camera System DP-72 (OLYMPUS, Tokyo, Japan) and differentially quantified by two pathologists who were from the Xiangya Hospital, Changsha, China.

GINS4 were considered positively by cytoplasmic expression. The determination results were obtained from semi-quantitative classification according to 10 or more visual fields (×200). The slides were first scored as 0 (negative), 1 (buff), 2 (pale brown), and 3 (tan). Positive expression of LSH was scored as 0 (negative), 1+ (<10% of positively-staining tumor cells), 2+ (11-50% of positively-staining tumor cells), 3+ (50-75% of positively-staining tumor cells), and 4+ (>75% of positively-staining tumor cells). Both the scores by multiply were regarded as the determination result.

**References**:

1. Shi Y, Tao Y, Jiang Y, Xu Y, Yan B, Chen X, et al. Nuclear epidermal growth factor receptor interacts with transcriptional intermediary factor 2 to activate cyclin D1 gene expression triggered by the oncoprotein latent membrane protein 1. Carcinogenesis 2012;33(8):1468-78.

2. Jiang Y, Yan B, Lai W, Shi Y, Xiao D, Jia J, et al. Repression of Hox genes by LMP1 in nasopharyngeal carcinoma and modulation of glycolytic pathway genes by HoxC8. Oncogene 2015;34(50):6079-91.

**Supplementary Figures and Figure Legends**

**Figure S1**. GINS4 expression levels were significantly elevated in lung cancer tissues. Dot blot are shown for the expression levels of GINS4 in lung ADCs (A) and SCCs (B). ****P < 0.001

**
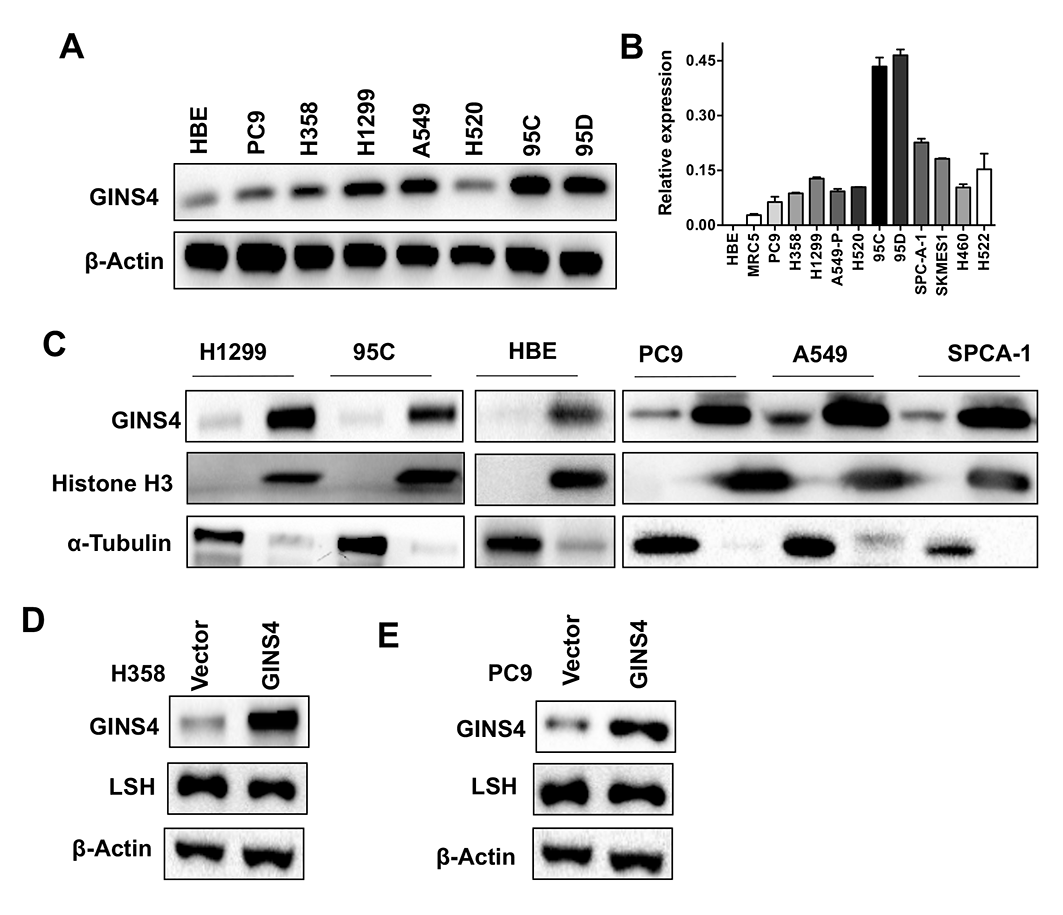
**

**Figure S2**. Stable GINS4 expression in lung cancer cells.

(A) Western blotting analysis and (B) qRT-PCR were performed to detect GINS4 expression in a panel of lung cancer cells. (C) Western blotting analysis was performed to detect GINS4 expression in the cytoplasmic and nuclear fractions of H1299, 95C, PC9, A549, SPCA-1 and HBE cells. (D and E) Western blotting analysis of GINS4 and LSH in GINS4-overexpressing (D) PC9 and (E) H358 cells.

Figure S3. Hematoxylin and Eosin staining, *ISH* for GIAT4RA and [immunohistochemistry](https://www.sciencedirect.com/topics/medicine-and-dentistry/immunohistochemistry) staining for the indicated proteins of a representative primary [xenograft](https://www.sciencedirect.com/topics/medicine-and-dentistry/xenograft) originating from GINS4-overexpressing PC9 (left panel) and H358 (right panel) cells or control cells. Original magnification 400 × , scale bar, 50 μm.


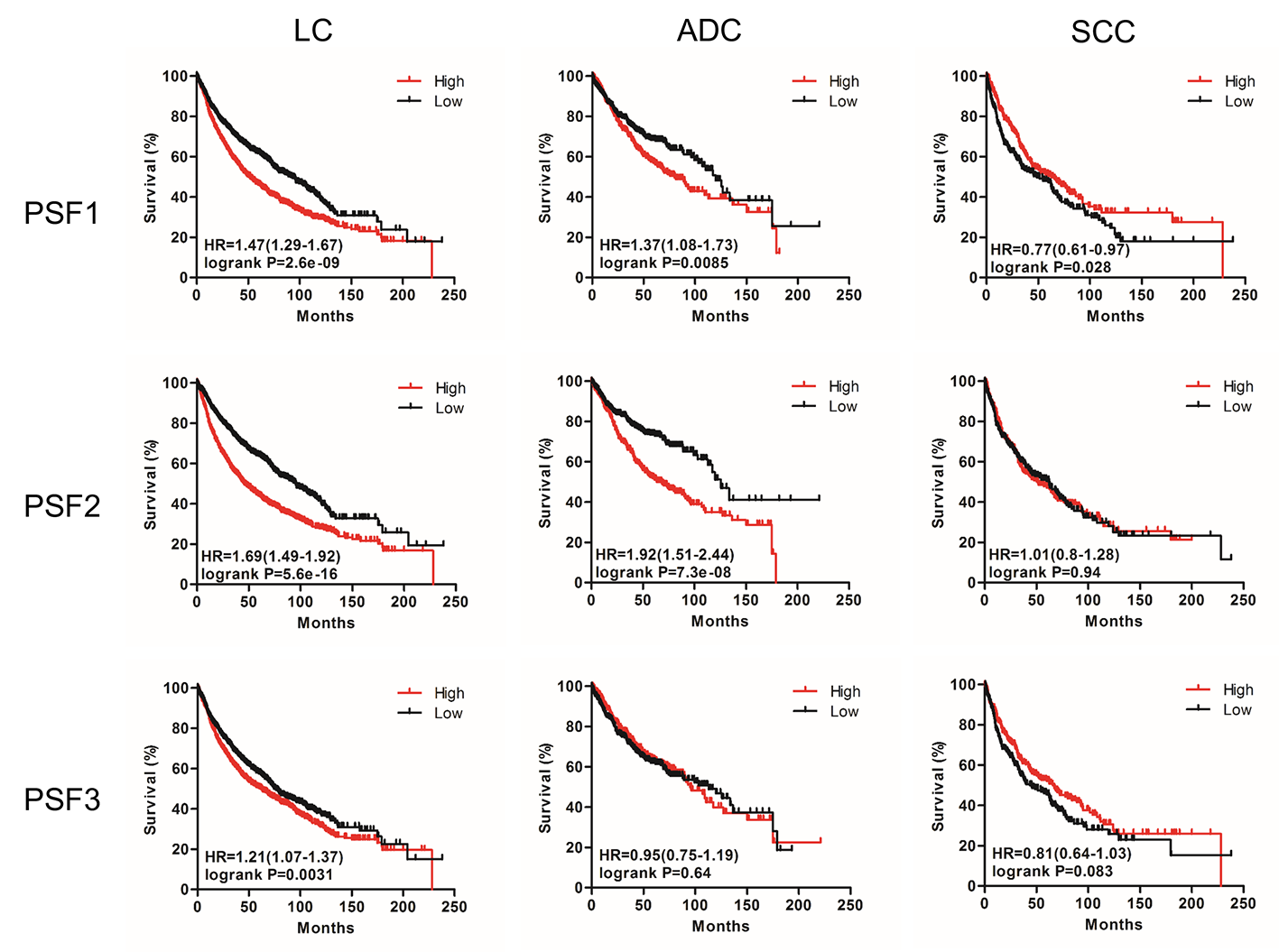


**Figure S4**. PSF1, PSF2, and PSF3 expression levels were significantly elevated in lung cancer tissues. Kaplan–Meier curves are shown for the overall survival rates of patients with lung cancer, ADCs, and SCCs.


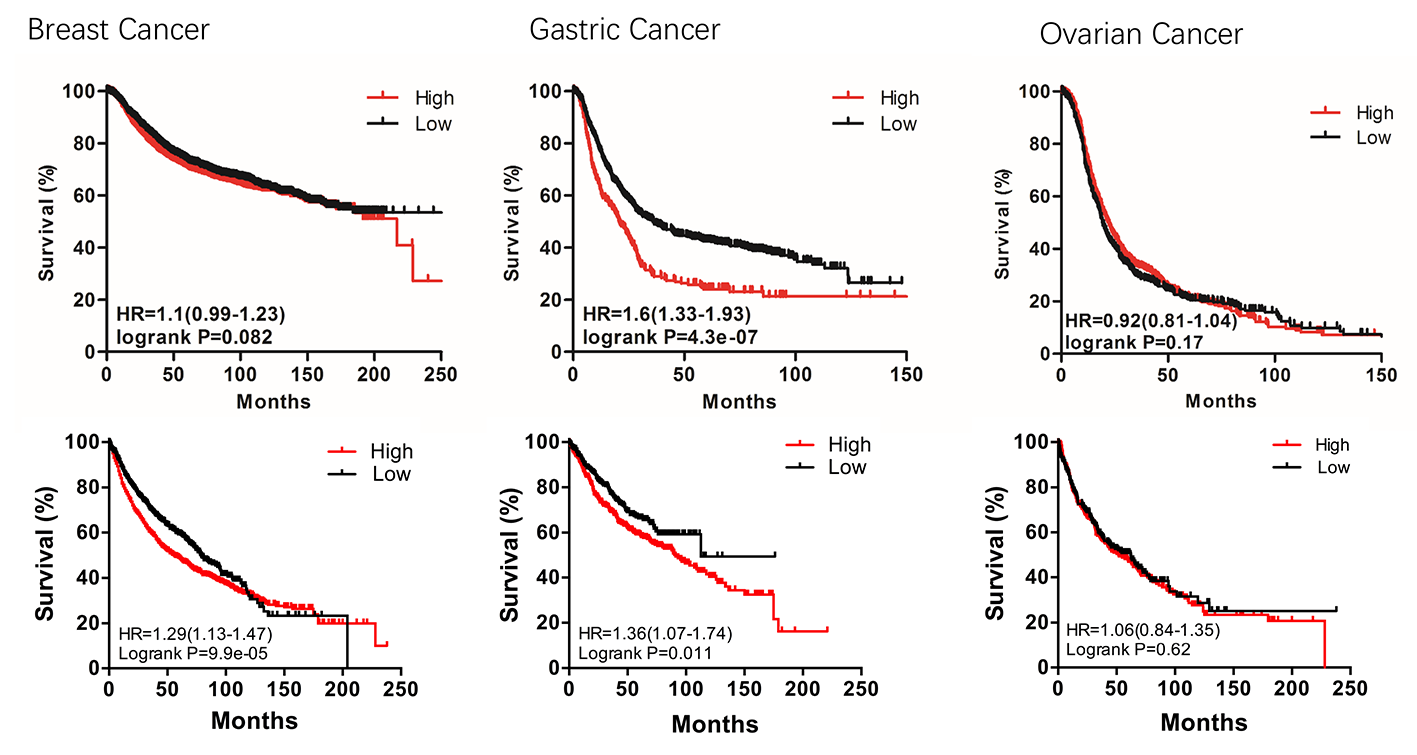


**Figure S5**. Elevated GINS4 expression levels in breast cancer, gastric cancer, and ovarian cancer.
